# Clinical features of a series of patients in life-threatening situations at a COVID-19 pandemic field hospital, evaluated by teleconsultation: evidence for Telemedicine expansion

**DOI:** 10.31744/einstein_journal/2021CE6370

**Published:** 2021-05-27

**Authors:** Tarso Augusto Duenhas Accorsi, Alexandra Régia Dantas Brigido, Karine De Amicis, Deborah de Sá Pereira Belfort, Fábio Cetinic Habrum, Fernando Garcia Scarpanti, Iuri Resedá Magalhães, José Roberto de Oliveira Silva, Leon Pablo Cartaxo Sampaio, Maria Tereza Sampaio de Sousa Lira, Renata Albaladejo Morbeck, Carlos Henrique Sartorato Pedrotti, Fábio de Castro Jorge Racy, Eduardo Cordioli

**Affiliations:** 1 Hospital Israelita Albert Einstein São PauloSP Brazil Hospital Israelita Albert Einstein, São Paulo, SP, Brazil.; 2 Instituto Israelita de Responsabilidade Social Hospital Israelita Albert Einstein São PauloSP Brazil Instituto Israelita de Responsabilidade Social, Hospital Israelita Albert Einstein, São Paulo, SP, Brazil.

Dear Editor,

Coronavirus disease (COVID-19) patients who are admitted due to respiratory distress may present gastrointestinal bleeding and thrombotic events, as well as renal, cardiac, hepatic and neurologic complications.^(1)^ These patients require advanced medical resources and longer length of hospital stay, leading to overburdened hospitals and emergency field hospital construction with massive physician hiring.^(2)^ In this scenario of human resources scarcity and high risk of transmission, Telemedicine has been a useful tool, helping healthcare providers properly manage complex cases despite the frontline medical team heterogeneity. Telemedicine provides excellent access to subspecialists, whose assessment can be crucial in the patient care process.^(3)^ Although the COVID-19 pandemic has escalated the use of Telemedicine worldwide and its benefits have been described in multiple medical settings, including disasters and public health emergencies, no study has analyzed the clinical features and interventions of Telemedicine-evaluated patients in life-threatening situations, at a field hospital.^(4)^

This study addresses a case series of severely-ill adult patients admitted to the *Hospital de Campanha do Pacaembu* (São Paulo, SP, Brazil). All attending physicians had access to the *Hospital Israelita Albert Einstein* (HIAE) Telemedicine Center, at São Paulo (SP, Brazil), through dedicated hardware and requested on-demand teleconsultations to senior emergency physicians. Retrospectively, 16 consecutive adult patients (>18 years) were enrolled between April and June 2020. The inclusion criteria were patients presenting deterioration of clinical status due to dyspnea or hypotension. The complete clinical and test data were compiled for eligible patients. We excluded patients with internet connection problems. All statistics are descriptive only.

The population was predominantly female (56.2%), with a mean age of 67.2±7.6 years. Patients were admitted after 9.1±3.5 days of symptoms and stayed for 10.4±4.2 days at the hospital. Fifteen patients were submitted to COVID-19 or imaging tests for confirmation. Pre-existing comorbidities possibly related to poor prognosis were as follows: hypertension (81.3%), age older than 65 years (56.3%), diabetes (43.8%), obesity (6.3%), asthma (6.3%), smoking (18.8%), chronic obstructive pulmonary disease (6.3%), cerebrovascular disease (6.3%), chronic kidney disease (6.3%), immunosuppression (6.3%) and pregnancy (0%). Most patients presented cough (93.8%) and dyspnea (81.3%), but only 6.3% had persistent fever.

According to the local attending physicians’ clinical judgment, Telemedicine was triggered for specialist evaluation if the patient was facing severe clinical deterioration. Interventions guided or discussed via Telemedicine are listed in table 1. All patients were evaluated by real-time video conference with a local attending physician and a senior emergency physician, available 24x7, at the Telemedicine Center. In all cases, consultants advised initiating or expanding antibiotic therapy. In ten cases (62.5%), consultants guided point-of-care ultrasound, and few patients were prescribed vasopressors, vasodilators, neuromuscular blockers and corticosteroids. Step-by-step, invasive ventilation placement was performed for two patients (12.5%).

**Table 1:** Interventions guided or discussed by Telemedicine

Variable	Description
	n (%)
Antibiotics	16 (100)
Corticosteroids	3 (18.75)
Vasopressors	3 (18.75)
Vasodilators	1 (6.25)
Neuromuscular blockers	2 (12.5)
Non-invasive ventilation	2 (12.5)
Point-of-care ultrasound	10 (62.5)

Therefore, the present case series shows that emergency care video consultation can be a viable solution in pandemic situations at field hospitals, despite potential communication challenges. Another study also presented evidence that video call contact is possible even in critical conditions, such as sepsis and post-cardiac arrest care.^(5)^ In the given cohort, the remote specialists were only called upon in case of high-complexity clinical scenarios; even though, relevant interventions could be advised, potentially saving lives.

Telemedicine is a reality in health services, reducing medical intervention time and demonstrating high diagnostic accuracy and cost-effectiveness, thereby supporting the further improvement of Telemedicine solutions in these domains.^(6)^ Considering the data presented, senior emergency physician teleconsultation for severely-ill patients can be a feasible and effective alternative to an *in-situ* evaluation in pandemic situations.
