# Supplementary material for: Clinical features of a series of patients in life-threatening situations at a COVID-19 pandemic field hospital, evaluated by teleconsultation: evidence for Telemedicine expansion
Source: Einstein (Sao Paulo). 2021 May 27;19:eCE6370. doi: 10.31744/einstein_journal/2021CE6370 (PMC8169026; doi:10.31744/einstein_journal/2021CE6370)
Supplement: Translation

# Translation: Características clínicas de uma série de pacientes críticos em um hospital de campanha durante a pandemia da COVID-19 avaliados por teleinterconsulta: evidência para expansão da Telemedicina

**Authors:** Tarso Augusto Duenhas Accorsi, Alexandra Régia Dantas Brigido, Karine De Amicis, Deborah de Sá Pereira Belfort, Fábio Cetinic Habrum, Fernando Garcia Scarpanti, Iuri Resedá Magalhães, José Roberto de Oliveira Silva, Leon Pablo Cartaxo Sampaio, Maria Tereza Sampaio de Sousa Lira, Renata Albaladejo Morbeck, Carlos Henrique Sartorato Pedrotti, Fábio de Castro Jorge Racy, Eduardo Cordioli

**Affiliations:** 1 Brasil Hospital Israelita Albert Einstein, São Paulo, SP, Brasil.; 2 Brasil Instituto Israelita de Responsabilidade Social, Hospital Israelita Albert Einstein, São Paulo, SP, Brasil.

Caro Editor,

Os pacientes com a doença causada pelo novo coronavírus (COVID-19) hospitalizados por insuficiência respiratória pode apresentar sangramento gastrintestinal e eventos trombóticos, além de complicações renais, cardíacas, hepáticas e neurológicas.^(1)^ Esses pacientes necessitaram de recursos médicos avançados e internação mais prolongada, resultando em hospitais sobrecarregados e construção de hospitais de campanha de emergência, implicando grande contratação de médicos.^(2)^ Nesse cenário de escassez de recursos humanos e alto risco de transmissão, a Telemedicina foi uma ferramenta útil, que ajudou os profissionais de saúde a conduzir os casos complexos de forma adequada, mesmo com uma equipe médica heterogênea, na linha de frente. A Telemedicina oferece acesso excelente a especialistas, cuja avaliação pode ser crucial no processo de cuidados do paciente.^(3)^ Apesar da pandemia de COVID-19 ter incrementado o uso da Telemedicina no mundo todo e de seus benefícios terem sido descritos em diversos cenários médicos, incluindo desastres e emergências de saúde pública, nenhum estudo analisou as características clínicas e as intervenções de pacientes avaliados por Telemedicina em situações de risco de vida em um hospital de campanha.^(4)^

Este estudo abordou uma série de casos de pacientes adultos gravemente enfermos internados no Hospital de Campanha do Pacaembu, em São Paulo (SP). Todos os médicos de plantão tinham acesso ao Centro de Telemedicina do Hospital Israelita Albert Einstein (HIAE), em São Paulo, por meio de *hardware* específico, e solicitavam teleinterconsultas com médicos emergencistas experientes, quando necessário. Retrospectivamente, 16 pacientes adultos (>18 anos) consecutivos foram incluídos, entre abril e junho de 2020. Os critérios de inclusão foram pacientes que apresentaram deterioração da condição clínica por dispneia ou hipotensão. Os dados clínicos e de exames completos foram compilados para os pacientes elegíveis. Foram excluídos os atendimentos com problemas de conexão com a internet. Todas as estatísticas são apenas descritivas.

A população era predominantemente feminina (56,2%), com média de idade de 67,2±7,6 anos. Os pacientes foram admitidos após 9,1±3,5 dias de sintomas e permaneceram por 10,4±4,2 dias no hospital. Quinze pacientes fizeram teste para COVID-19 ou exames de imagens para confirmação da doença. As comorbidades preexistentes possivelmente relacionadas a pior prognóstico foram hipertensão (81,3%), idade acima de 65 anos (56,3%), diabetes (43,8%), obesidade (6,3%), asma (6,3%), tabagismo (18,8%), doença pulmonar obstrutiva crônica (6,3%), doença cerebrovascular (6,3%), doença renal crônica (6,3%), imunossupressão (6,3%) e gestação (0%). A maioria dos pacientes apresentou tosse (93,8%) e dispneia (81,3%), mas apenas 6,3% tiveram febre persistente.

Conforme o raciocínio clínico dos médicos de plantão no local, o Centro de Telemedicina era acionado para avaliação por especialista caso o paciente apresentasse deterioração clínica grave. As intervenções orientadas ou discutidas por Telemedicina estão relacionadas na tabela 1. Todos os pacientes foram avaliados por videochamada em tempo real, com um médico de plantão no local e um médico emergencista, disponível por 24 horas, por 7 dias da semana, no Centro de Telemedicina. Em todos os casos, os médicos da Telemedicina aconselharam iniciar ou ampliar a antibioticoterapia. Em dez casos (62,5%), a teleinterconsulta orientou a ultrassonografia à beira leito, enquanto vasopressores, vasodilatadores, bloqueadores neuromusculares e corticoides foram prescritos para poucos pacientes. A ventilação invasiva foi instalada, passo a passo para dois pacientes (12,5%).

**Tabela 1:** Intervenções guiadas ou discutidas por Telemedicina

Variável	Descrição
	n (%)
Antibióticos	16 (100)
Corticoides	3 (18,75)
Vasopressores	3 (18,75)
Vasodilatadores	1 (6,25)
Bloqueadores neuromusculares	2 (12,5)
Ventilação não invasiva	2 (12,5)
Ultrassonografia à beira leito	10 (62,5)

Portanto, esta série de casos mostra que a teleinterconsulta para cuidados de emergência pode ser uma solução viável em situação de pandemia, nos hospitais de campanha, apesar dos potenciais desafios de comunicação. Outro estudo também apresentou evidências de que o contato por videochamada é possível, mesmo em condições críticas, como sepse e atendimento pós-parada cardíaca.^(5)^ Nesta coorte, os especialistas remotos foram chamados apenas em situações de alta complexidade e, mesmo assim, as intervenções relevantes puderam ser orientadas, potencialmente salvando vidas.

A Telemedicina já é uma realidade nos serviços de saúde, reduzindo o tempo de intervenção médica e demonstrando alta precisão diagnóstica, além de boa relação custo-efetividade, justificando mais melhorias das soluções de Telemedicina nesses serviços.^(6)^ Com base nos dados apresentados, a teleinterconsulta com emergencista experiente para pacientes gravemente enfermos pode ser uma alternativa factível e efetiva para a avaliação *in situ* em situações de pandemia.
